# Alternative splicing and co-option of transposable elements: the case of TMPO/LAP2α and ZNF451 in mammals

**DOI:** 10.1093/bioinformatics/btv132

**Published:** 2015-03-02

**Authors:** Federico Abascal, Michael L. Tress, Alfonso Valencia

**Affiliations:** Structural Biology and Biocomputing Programme, Spanish National Cancer Research Centre, Madrid 28029, Spain

## Abstract

**Summary**: Transposable elements constitute a large fraction of vertebrate genomes and, during evolution, may be co-opted for new functions. Exonization of transposable elements inserted within or close to host genes is one possible way to generate new genes, and alternative splicing of the new exons may represent an intermediate step in this process. The genes *TMPO* and *ZNF451* are present in all vertebrate lineages. Although they are not evolutionarily related, mammalian *TMPO* and *ZNF451* do have something in common—they both code for splice isoforms that contain LAP2alpha domains. We found that these LAP2alpha domains have sequence similarity to repetitive sequences in non-mammalian genomes, which are in turn related to the first ORF from a DIRS1-like retrotransposon. This retrotransposon domestication happened separately and resulted in proteins that combine retrotransposon and host protein domains. The alternative splicing of the retrotransposed sequence allowed the production of both the new and the untouched original isoforms, which may have contributed to the success of the colonization process. The LAP2alpha-specific isoform of *TMPO* (LAP2α) has been co-opted for important roles in the cell, whereas the *ZNF451* LAP2alpha isoform is evolving under strong purifying selection but remains uncharacterized.

**Contact**: mtress@cnio.es or valencia@cnio.es

**Supplementary information:** Supplementary data are available at *Bioinformatics* online.

## 1 Introduction

Vertebrate genomes, like those of other eukaryotes, are largely constituted by transposable elements (TEs)—up to two-thirds of the human genome according to a recent estimate (de Koning *et al.*, 2011). The evolutionary history of TEs runs parallel to that of the host genome. All life forms are endowed with mechanisms to defend against the threat posed by the activity and expansion of TEs. Despite their threatening character, many TEs have been co-opted for new functions during evolutionary history, in what is considered a form of domestication (Feschotte, 2008; Hoen and Bureau, 2012; Volff, 2006). Some TEs have contributed non-coding regulatory sequences, whereas others have given rise to new protein-coding genes.

Basically, there are two means of co-opting TEs into genes. First, a TE (or part of one) may form a new whole gene. This is probably what occurred with TERT, the telomerase of eukaryotes, which probably originated from the reverse transcriptase (RT) of a non-LTR retrotransposon (Lingner *et al.*, 1997). Alternatively, a TE can insert within or close to a pre-existing gene and combine with it to produce a new protein. This is what happened in the case of the histone methyltransferase SETMAR in the ancestor of primates. Here, a mariner-like DNA transposon inserted downstream of the SET CDS producing an exon that combined with the original exons to form a chimeric protein (SETMAR) with a new DNA-binding domain (Cordaux *et al.*, 2006).

The process of TE exonization has been studied thoroughly (Cordaux and Batzer, 2009; Sela *et al*., 2010; Sorek, 2007). Many TE-derived exons have been identified in the human genome, the majority are fragments of Alu elements (Deininger, 2011). Most Alu-derived exons are alternatively spliced, presumably because their constitutive expression would be deleterious and is negatively selected (Sorek *et al.*, 2002). It has been proposed that the exonization of TEs would have less deleterious effects if the exons were alternative (Schmitz and Brosius, 2011; Xing and Lee, 2006), because they would not disturb the production of the original isoforms and alternative splicing would allow TEs to coexist in an intermediate stage on the way to full TE co-option.

Most new TE exons appear in 5- or 3-prime UTRs and may play regulatory roles (Sela *et al.*, 2007; Shen *et al.*, 2011). In contrast, TE exonization in protein-coding regions generally produces short, lineage-specific exons that appear as rare splice variants, questioning their biological relevance and suggesting that most probably do not translate into proteins (Gotea and Makałowski, 2006; Lin *et al*., 2008; Pavlíček et al., 2002; Piriyapongsa *et al*., 2007).

Here, we investigate the likely TE origins of exons found in both the *ZNF451* and *TMPO* genes. These exons are found in the coding regions of both genes, are alternatively spliced and there is ample evidence of their expression as proteins. The exons are present in mammalian orthologs of *ZNF451* and *TMPO*, whereas they are absent in other vertebrates.

## 2 Methods

To identify genes with mutually exclusive protein domains, we retrieved human transcripts from Ensembl (Cunningham *et al.*, 2014) that were annotated with CCDS support (Pruitt *et al.*, 2009). We took their Pfam annotations (Finn *et al.*, 2014) and looked for alternative transcripts with different Pfam domains that were mutually exclusively spliced. We identified 40 candidates, of which only nine were retained after manual curation: *MOCS2*, *DST*, *XIRP2*, *CUX1*, *SP100*, *ZNF655*, *GNAS*, *VTN* and *ZNF451*. Proteomics experiments identified peptide support for alternative isoforms for four of these genes (Ezkurdia *et al.*, 2012).

Sequence similarity searches between the LAP2alpha domains (from the *TMPO* and *ZNF451* genes) and vertebrate genome sequences were carried out using TBLASTN, via the Ensembl web server (Cunningham *et al.*, 2014) or locally, in both cases using version 2.2.26 of the BLAST package (Altschul *et al.*, 1997). RPS-TBLASTN profile-based searches (v2.2.29) were performed against the CDD database [version of October 2014 (Marchler-Bauer *et al.*, 2005)]. The Western painted turtle genome [v3.0.1 (Shaffer *et al.*, 2013)] was downloaded from UCSC (Rosenbloom *et al.*, 2014). Repeat annotations for the Chinese soft-shell turtle were obtained from Ensembl, including RepeatMasker (Smit *et al.*, 1996) and RepeatModeler (Smit and Hubley, 2010) predictions. Sequences from genomic regions were extracted with bedtools v2.17.0 (Quinlan and Hall, 2010). Multiple sequence alignments were built with Mafft v6.8.57 (Katoh and Standley, 2013), through Jalview v14 (Waterhouse *et al.*, 2009). The maximum-likelihood phylogenetic tree was built with Phyml v3 (Gouy *et al*., 2010; Guindon *et al*., 2010) using the best-fit evolutionary model HIVb (Nickle *et al.*, 2007) with gamma-rate heterogeneity and empirical amino acid frequencies (HIVb+G+F), as determined with ProtTest v2.4 (Abascal *et al.*, 2005). Independent multiple sequence alignments were built for the respective LAP2alpha domains of LAP2α and ZNF451-L2a. Overall non-synonymous by synonymous substitution rates were calculated for each of these alignments using the SLAC method (Kosakovsky Pond and Frost, 2005) available in the DataMonkey web server (Delport *et al.*, 2010).

## 3 Results

### 3.1 Two genes and an alternatively spliced mammalian-specific protein domain

**Fig. 1.**
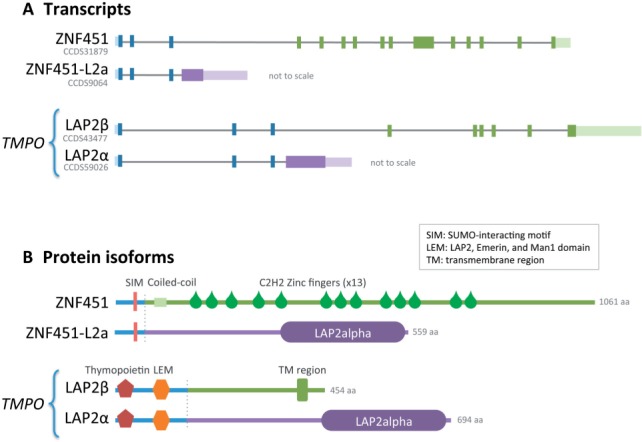
Schematic organization of the *ZNF451* and *TMPO* transcripts (**A**) and the domain organization of the translated protein isoforms (**B**). The presence of a LAP2alpha domain within ZNF451-L2a is supported by an Hmmscan search against Pfam (*e* value of 1.5e-08). The LAP2alpha domain of both *TMPO* and *ZNF451* is coded by a large 3-prime exon. The exon includes a polyadenylation signal that prevents the simultaneous incorporation of other exons. In the case of *ZNF451*, alternatively skipped 3-prime exons code for multiple Zinc fingers. In the case of *TMPO*, they code for the C-terminal half of the protein, which includes a nuclear membrane attachment trans-membrane helix. The exon coding the LAP2alpha domain translates into amino acid regions of 497 and 506 residues (9114 and 2812 bp) in *ZNF451* and *TMPO*, respectively

Very few human protein-coding genes generate alternative isoforms in which one Pfam functional domain (Finn *et al*., 2014) is substituted for another. *ZNF451* is one of just nine of these genes. One of the two *ZNF451* splice isoforms has a single large 3′-exon with a polyadenylation signal that codes for a C-terminal ‘LAP2alpha’ domain. The incorporation of the polyadenylation signal prevents the incorporation of downstream exons coding for multiple zinc fingers (Fig. 1). The LAP2alpha domain is found in just one other human gene, *TMPO*. *TMPO* has a similar pattern of alternative splicing to *ZNF451*, here the LAP2alpha C-terminal domain replaces a protein region that contains a nuclear attachment trans-membrane (TM) region (Fig. 1). The *TMPO* LAP2alpha isoform is known as LAP2α, whereas the *ZNF451* isoform is referred to as ZNF451-L2a in this work.

*TMPO* (lamina-associated polypeptide 2 gene) codes for several protein isoforms, the most studied of which are LAP2β and LAP2α, with marked differences in their cellular roles (Berger *et al*., 1996; Dechat *et al*., 2000). The LAP2β isoform is conserved as far back as vertebrates and can attach to the nuclear envelope through its C-terminal TM region and bind lamin B to the nuclear lamina (Foisner and Gerace, 1993). *ZNF451* also evolved along with vertebrates. The main isoform of *ZNF451* is a nuclear protein that, upon sumoylation, localizes to promyelocytic leukemia bodies and interacts with the androgen receptor (Feng *et al*., 2014; Karvonen *et al*., 2008).

Interestingly, although the genes *TMPO* and *ZNF451* are present in all vertebrate lineages, the isoforms containing the LAP2alpha domain are exclusive to mammals (*TMPO*) or eutherians (*ZNF451*). This observation is intriguing and prompted us to determine the origin of the LAP2alpha domain and why it is conserved in mammals.

### 3.2 The LAP2alpha domain originated from the GAG ORF of a DIRS1-like retrotransposon

To characterize the origin of the LAP2alpha domain, we searched vertebrate genomes using TBLASTN with the amino acid sequences of the LAP2alpha domains of ZNF451-L2a and LAP2α. In placental mammals, we found homology in just *TMPO* and *ZNF451*. There was no significant homology within the *ZNF451 locus* in opossum or platypus, and no significant similarity was found within the *TMPO* and *ZNF451* loci in any non-mammalian vertebrate.

**Fig. 2.**
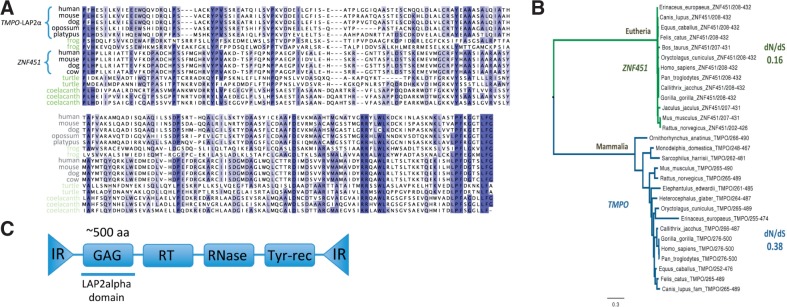
(**A**) A multiple sequence alignment including the LAP2alpha domains of mammalian LAP2α and ZNF451-L2a and homologous sequences from non-mammalian DIRS1-like elements. The phylogenetic tree in (**B**) was built with Phyml for the LAP2alpha domains of LAP2α and ZNF451-L2a. Overall dN/dS ratios were calculated with the SLAC method in DataMonkey (see Supplementary Appendix). (**C**) A schematic structure of the DIRS1-like element from which the LAP2alpha domain originated

In contrast, we found numerous significant similarity hits (TBLASTN *e* value < 1e-05) within the genomes of anole lizard (6), Chinese soft-shell turtle (9) and coelacanth (11; Fig 2A). With further sequence searches, we found that these genomes in fact contained hundreds or thousands of copies (TBLASTN hits), revealing the repetitive nature of these sequences and suggesting a TE origin.

These repetitive sequences are only annotated in Chinese soft-shell turtle [*Pelodiscus sinensis* (Cunningham *et al*., 2014; Wang *et al*., 2013)]. Within the turtle, most of the TBLASTN similarity hits overlapped or were very close to repeats annotated as DIRS1-like, suggesting that the LAP2alpha domain may have originated from a DIRS1-like element.

The family of DIRS1 elements has been found in almost all eukaryotes, and species-specific expansions have been reported in anole lizard, *Xenopus* and zebrafish (Goodwin and Poulter, 2001; Piednoël *et al*., 2011). DIRS1-like elements are related to LTR-retrotransposons and retroviruses, but they have distinctive features (Cappello *et al*., 1985)—the presence of inverted terminal repeats (ITRs instead of LTRs) and a specific mechanism for genome integration based on the action of a tyrosine recombinase related to bacteriophage integrases (Goodwin and Poulter, 2001).

To assess the hypothesis of a DIRS1-like origin for the mammalian LAP2alpha domain, we looked for evidence in addition to the repeat annotations from the Chinese soft-shell turtle. We took 792 genomic regions from the Western painted turtle [*Chrysemys picta bellii plus* (Shaffer *et al*., 2013)] with homology to the LAP2alpha domain plus 5000 downstream and upstream bps. These regions were scanned with RPS-TBLASTN against the CDD database (Marchler-Bauer *et al*., 2005). We found sequences coding for RTs, RNases and phage integrases downstream to the regions homologous to LAP2alpha domains, in most cases of the subtype RT_DIRS1 and RNase_HI_RT_DIRS1 (642 and 594 cases, respectively), which are characteristic of DIRS1-like elements. We also identified domains typical of phage-integrases, which are related to the tyrosine recombinase activity of DIRS1-like elements (Goodwin and Poulter, 2001). We manually analyzed some sequences and confirmed the presence of ITRs instead of LTRs, characteristic of DIRS1-like elements (Fig. 2C). Hence, we confirmed that the LAP2alpha domain is related to DIRS1-like elements.

DIRS1-like retrotransposons are composed of two ITRs and four ORFs (Fig. 2C). The first ORF is usually referred to as GAG based on the order of ORFs found in related LTR-retrotransposons and retroviruses, where the GAG ORF codes for proteins of the nucleocapsid. However, no clear homology between ORF1 of DIRS1-like elements and known retroviral GAG proteins has been found (Goodwin and Poulter, 2001). The second ORF codes for an RT, whereas the third and fourth ORFs code for an RNase and a tyrosine-recombinase (Goodwin and Poulter, 2001). We analyzed the relative position of regions similar to RNases, RTs and phage-recombinases with respect to the region found to be homologous to the LAP2alpha domain in the Western painted turtle. Our results indicate that the LAP2alpha domain is homologous to the GAG gene, the first ORF of DIRS-1-like elements.

### 3.3. Functional implications of the LAP2alpha domain domestication

There is peptide evidence for the expression of both *TMPO* and *ZNF451* LAP2alpha isoforms in mass spectrometry experiments (F. Abascal *et al.*, submitted). Moreover, LAP2α is the alternative splice isoform that has most peptide support.

The *TMPO* LAP2alpha isoform has important cellular roles in mammals. LAP2α binds lamin A in the nucleoplasm (Dechat *et al*., 2000; Naetar *et al*., 2008) and, together with lamin A/C, binds unphosphorylated Rb, tethering it to the nucleus (Markiewicz *et al*., 2002). This allows Rb to control cell-cycle progression (Giacinti and Giordano, 2006). Interestingly, LAP2α is found within the retroviral pre-integration complex and is necessary for the integration of the Moloney murine leukemia virus (Suzuki *et al*., 2004), which may reflect the ancestral role of the LAP2alpha domain. A mutation in the LAP2alpha domain can cause dilated cardiomyopathy (Taylor *et al.*, 2005).

The function of the ZNF451-L2a isoform has not been characterized. Because the sumoylation motif falls within the N-terminus that is shared between *ZNF451* isoforms, ZNF451-L2a may be also a target of sumoylation. The functional relevance of ZNF451-L2a is supported by the observation that its LAP2alpha domain is evolving under strong purifying selection, even stronger than in LAP2α (overall dN/dS of 0.16 for ZNF451-L2a and 0.38 for LAP2α, see Section 2; Fig. 2B).

## 4 Conclusions

The evidence presented here strongly supports the possibility that the LAP2alpha domain present in *TMPO* and *ZNF451* has a retrotransposon origin that can be traced back to a DIRS1-like element and the ORF1 (GAG) gene in particular. We have found both direct and indirect supporting evidence for this. Indirect evidence includes (i) LAP2alpha domains are coded by single large exons, a hallmark of retroposition; (ii) although the genes *TMPO* and *ZNF451* are present in other vertebrates, the LAP2alpha domain coding exon is specific to mammals and (iii) in both genes, the mammalian-specific exon is subject to alternative splicing. Direct evidence relies on the very significant sequence similarity between the LAP2alpha domain of both LAP2α and ZNF451-L2a and ORF1 from DIRS1-like retro-TEs.

Many cases of domestication of TEs that have been co-opted for new functions in the cell have been reported (Volff, 2006). In the case presented here, the integration of the DIRS1-like element within pre-existing genes has given rise to chimeric proteins in which the LAP2alpha domain has replaced other protein domains. These new proteins have been maintained throughout the evolution of mammals and are evolving under strong purifying selection, supporting their functional relevance. Probably, the most remarkable feature of this case is that alternative splicing allowed both the new and the original isoforms to coexist. Based on the observation that the same pattern of alternative splicing is found within the *ZNF451* and *TMPO* genes, we infer that the colonizing sequence probably carried an alternative splice acceptor site plus a polyadenylation signal. This could be seen as a neutral colonization that avoided disrupting the original isoforms and making possible the production of new alternative isoforms. This characteristic may have contributed to LAP2alpha’s successful double colonization of two independent genes.

## Funding

This work was supported by grants NIH [U41 HG007234] and Spanish MINECO [grant Bio2012-40205].

*Conflict of Interest*: none declared.

## Supplementary Material

Supplementary Data
