# Supplementary material for: Alternative splicing and co-option of transposable elements: the case of TMPO/LAP2α and ZNF451 in mammals
Source: Bioinformatics. 2015 Mar 2;31(14):2257–61. doi: 10.1093/bioinformatics/btv132 (PMC4495291; doi:10.1093/bioinformatics/btv132)
Supplement: Supplementary Data [file supp_31_14_2257__index.html]

Alternative splicing and co-option of transposable elements: the case of TMPO/LAP2α and ZNF451 in mammals — Alternative splicing and co-option of transposable elements: the case of TMPO/LAP2α and ZNF451 in mammals — Supplementary Data 

# Alternative splicing and co-option of transposable elements: the case of TMPO/LAP2α and ZNF451 in mammals

## Supplementary Data

files

**Files in this Data Supplement:**

- Supplementary Data - pdf file
- Supplementary Data - xlsx file
