# Supplementary material for: Alternative splicing and co-option of transposable elements: the case of TMPO/LAP2α and ZNF451 in mammals
Source: Bioinformatics. 2015 Mar 2;31(14):2257–61. doi: 10.1093/bioinformatics/btv132 (PMC4495291; doi:10.1093/bioinformatics/btv132)
Supplement: Supplementary Data [file supp_btv132_Appendix.pdf]

Appendix

1. Sequence analysis of the Chinese soft-shell turtle (*Pelodiscus sinensis*) genome
2. Sequence analysis of the Western painted turtle (*Chrysemys picta bellii plus*) genome
3. Strength of purifying selection acting on the LAP2alpha domain of the genes *ZNF451* and *TMPO/LAP2α*

Sequence analysis of the Chinese soft-shell turtle (*Pelodiscus sinensis*) genome

Sequence searches were conducted with the amino acid sequences of the human *TMPO* (LAP2α) and *ZNF451* genes corresponding to the alternative exons that code for the LAP2alpha domain: Uniprot accession number P42166 (residues 189-694) for *TMPO* (LAP2α), and Ensembl identifier ENSP00000359742/ENST00000370708 (residues 63-559) for *ZNF451* (the ZNF451-L2a isoform has not been annotated yet in the Uniprot entry corresponding to human *ZNF451*, Q4KMR5).

A TBLASTN search against the Chinese soft-shell turtle (*Pelodiscus sinensis*) genome with the human LAP2alpha sequences identified 835 non-overlapping significantly similar hits (e-value < 0.01). We determined the overlap between these TBLASTN hits (+/- 200 flanking bps) and repeats annotated with RepeatMasker and RepeatModeler in Ensembl. Up to 734 hits overlapped or were close to repeats annotated as DIRS (see supplementary tables). Within the genome of *P. sinensis* there are 37,215 repeats annotated as DIRS.

Supplementary table 1.

Overlap between TBLASTN hits and annotated repeats *		
**Repeat**	**Number of overlaps**	**Number of overlaps +/-200 bps**
DIRS / LTR	570	734
Penelope / LINE	163	193
Unknown	87	144
Copia / LTR	55	68

Sequence analysis of the Western painted turtle (Chrysemys picta bellii plus)

A TBLASTN search within the Western painted turtle genome using human LAP2alpha query sequences identified 830 non-overlapping significantly similar hits. We obtained the corresponding genomic sequence for each of these hits, either adding 200 or 5,000 flanking bps to investigate what was in the neighborhood of each of the TBLASTN hits. These nucleotide sequences were compared to protein profiles available in the CDD database using RPS-TBLASTN, either with the SEG filter on or off, and setting the e-value threshold at 0.01.

For the set of sequences with +/- 200 flanking bps, the most frequent CDD hit corresponded to the LAP2alpha domain described in Pfam, as expected. The fact that only 59 (or 135 if the SEG filter was turned off) out of 830 sequences were found significantly similar to the LAP2alpha profile is related to how this Pfam signature was built. The Pfam LAP2alpha domain profile was trained with

LAP2alpha domains from *TMPO* (LAP2α) proteins, not including *ZNF451* proteins. Indeed, many more sequence similarity hits within the turtle genome were found with the LAP2alpha domain of *ZNF451* (821) than with the LAP2alpha domain of *TMPO* (100).

Supplementary table 2.

RPS-TBLASTN search of TBLASTN hits +/- 200 bps vs CDD profiles (e-value < 0.01, seg=yes)		
**Family**	**Number of hits**	**Description**
**LAP2alpha**	59	Lamina-associated polypeptide 2 alpha
PHA03247	3	Large tegument protein UL36 (Herpes simplex virus)

We explored the set of sequences with +/-5,000 flanking bps to determine which other protein profiles are found in the neighborhood of sequences similar to the LAP2alpha domain. Our results show that reverse transcriptase and RNase_H from DIRS1 elements are the most frequent and most significant hits. Interestingly, we also found similarities to other protein profiles that are characteristic of viruses and retrotransposons (supplementary table 3).

Supplementary table 3.

RPS-TBLASTN search of TBLASTN hits +/- 5,000 bps vs CDD profiles (e-value < 0.01, seg=yes)		
**Family**	**Number of hits**	**Description**
**RT_DIRS1**	641	Reverse transcriptases (RTs) occurring in the DIRS1 group of retransposons
RT_LTR	602	Reverse transcriptases (RTs) from retrotransposons and retroviruses which have long terminal repeats (LTRs) in their DNA copies but not in their RNA template
**RNase_HI_RT_DIRS1**	594	DIRS1 family of RNase HI in long-term repeat retroelements
RVT_1	566	Reverse transcriptase (RNA-dependent DNA polymerase)
RT_Rtv	449	Reverse transcriptases (RTs) from retroviruses (Rtvs)
RT_ZFREV_like	370	A subfamily of reverse transcriptases (RTs) found in sequences similar to the intact endogenous retrovirus ZFERV from zebrafish and to Moloney murine leukemia virus RT
RNase_HI_RT_Ty3	204	Ty3/Gypsy family of RNase HI in long-term repeat retroelements
PHA03247	158	Large tegument protein UL36 (Herpes simplex virus)
RT_nLTR_like	110	Non-LTR (long terminal repeat) retrotransposon and non-LTR retrovirus reverse transcriptase (RT)
Phage_int_SAM_1	103	Phage integrase, N-terminal SAM-like domain.
PHA03307	89	Transcriptional regulator ICP4 (Herpes simplex virus)
RT_like	82	Reverse transcriptase (RT, RNA-dependent DNA polymerase)_like family
recomb_XerD	73	Tyrosine recombinase XerD (phage)
PRK07764	61	DNA polymerase III subunits gamma and tau
RT_pepA17	52	Reverse transcriptase (RTs) in retrotransposons
**LAP2alpha**	51	Lamina-associated polypeptide 2 alpha
RT_G2_intron	49	Reverse transcriptases (RTs) with group II intron origin
XerD	39	Site-specific recombinase XerD [DNA replication, recombination, and repair]
Atrophin-1	39	Atrophin-1 family
xerD	35	Site-specific tyrosine recombinase XerD
Tymo_45kd_70kd	35	Tymovirus 45/70Kd protein
PHA03378	34	EBNA-3B (Epstein-Barr virus)
Phage_int_SAM_4	30	Phage integrase, N-terminal SAM-like domain

Strength of purifying selection on the LAP2alpha domain of the genes *ZNF451* and *TMPO* (LAP2α)

To measure the strength of purifying selection, we concentrated on the region corresponding to the LAP2alpha domains of the LAP2α and ZNF451-L2a isoforms, as this is clearly more conserved than the rest of the corresponding alternative exons. Because the two isoforms are highly divergent, we build independent multiple sequence alignments for each of the two genes, using the same species sampling. Then, we measured the strength of purifying selection for each of these two alignments. We tested two methods for calculating the overall ratios between non-synonymous and synonymous substitution rates (dN/dS), obtaining similar results. The SLAC method available in the DataMonkey web-server estimated dN/dS ratios of 0.38 (0.34 according to PAML/Codeml) and 0.16 (0.14) for the LAP2alpha domains of LAP2α and ZNF451-L2a, respectively. This indicates that the LAP2alpha domain of ZNF451 is evolving under stronger purifying selection. Indeed, the corresponding multiple alignments clearly show that the LAP2alpha domain of *ZNF451* has been conserved to a greater extent than in *LAP2α.*
